# Introducing the 3^rd^ Annual PARP and DNA Damage Response Inhibitors Summit

**DOI:** 10.36401/2590-017X-3-1.e8

**Published:** 2020-03-31

The *Journal of Immunotherapy and Precision Oncology* (JIPO) is proud to support this event and encourages readers to attend in Boston, MA on January 28–30, 2020.

As the field continues to evolve at an accelerated rate, it is important for the industry to gather and discuss their challenges and share their solutions. Industry-focused meetings such as the **PARP and DDR Inhibitors Summit** (Jan 28–30, 2020 in Boston, MA, previously “DNA Damage Response Therapeutics Summit”) are key events to meet the *stars* of your DDR news feeds! The 3-day event is designed to equip researchers and drug developers with the tools for blockbuster therapy success and the connections to effectively advance their work.

The meeting boasts 20 plenary sessions, 25 expert speakers and 2 open panel discussions. Experts from Cybrexa Therapeutics, Cyteir and Ribon Therapeutics will be there to run analysis of 7 novel DDR targets in addition to 2 exclusive deep-dive workshops. Attendees will take advantage of more than 5 hours of networking with 80+ leaders in the space. The major challenges and successes for DDR researchers will be addressed by the Heads, Chiefs, and Presidents of discovery, translation, and clinical practice from Pharma Biotech and Academia.

## An Exclusive Interview with JIPO Editor and Speaker, Dr. Timothy Yap

Dr. Timothy Yap is a Medical Oncologist and Physician-Scientist based at the University of Texas MD Anderson Cancer Center.

Dr. Yap's main research focuses on the first-in-human and combinatorial development of molecularly targeted agents and immunotherapies, and their acceleration through clinical studies using novel predictive and pharmacodynamic biomarkers.

### What, in your opinion, are the 3 greatest challenges faced in the DDR therapy field today?

- Determining the specific mechanisms that underlie response and resistance to DDR therapies
- Establishing registration strategies for DDR therapies beyond PARP inhibitors, e.g. ATR, DNA-PK, ATM, WEE1 inhibitors.
- Managing challenging overlapping toxicities observed with DDR combination therapies, e.g. myelosuppression.

### How do you envision these challenges being addressed? What interesting progress being made to further the field?

Translational studies undertaken on sequential tumor biopsies and ctDNA samples taken from patients on DDR therapies will provide greater insights into specific mechanisms that underlie response and resistance to DDR therapies

Innovative early phase clinical trials that assess the respective roles of DDR therapies beyond PARP inhibitors are being undertaken with early promise being made.

Innovative strategies, such as the creative scheduling of DDR combination therapies, as well as the use of drug conjugates with alphalex peptides to enable a more tumor-specific delivery of DDR agents are showing promise preclinically, and are novel approaches about to enter the clinic soon.

### What are the most important or promising DDR targets in your opinion and why?

The most important DDR targets are PARP, ATR, ATM, DNA-PK, WEE1, CHK1/2 and POLQ, as these are potentially druggable targets for the clinic.

### Where do you see DDR therapy fitting in the universe of cancer therapy? ie combination approaches, maintenance therapy, which line of therapy, precision medicine…

I see DDR therapy as a platform therapy with multiple applications, as monotherapy using molecularly-driven approaches, as seen with BRCA1/2 mutant cancers, both in the maintenance and relapse settings, in differrernt lines of therapy, and also in combination approaches to deepen responses, reverse resistance and increase the proportion of patients who may respond to single agent strategies.

